# Supplementary material for: Hecaton: reliably detecting copy number variation in plant genomes using short read sequencing data
Source: BMC Genomics. 2019 Nov 7;20:818. doi: 10.1186/s12864-019-6153-8 (PMC6836508; doi:10.1186/s12864-019-6153-8)
Supplement: Supplementary file 2 — Additional file 2 Table S1: Features used for the random forest model. Table S2: Description of the used datasets. Table S3: Number of events called from B73 data that could not be validated by VaPoR. Table S4: Number of events simulated per size interval. [file 12864_2019_6153_MOESM2_ESM.pdf]

Table S1: **Features used for the random forest model**

Feature	Type	Description
READ_PAIRS	Integer	Number of supporting discordantly aligned reads pairs
SPLIT_READS	Integer	Number of supporting split reads
SIZE	Integer	Size of the event in bp
DELLY	Binary	1 if event is supported by Delly, 0 otherwise
GRIDSS	Binary	1 if event is supported by GRIDSS, 0 otherwise
LUMPY	Binary	1 if event is supported by LUMPY, 0 otherwise
MANTA	Binary	1 if event is supported by Manta, 0 otherwise
DEL	Binary	1 if event is a deletion, 0 otherwise
INS	Binary	1 if event is an insertion, 0 otherwise
TANDUP	Binary	1 if event is a tandem duplication, 0 otherwise
DISDUP	Binary	1 if event is a dispersed duplication, 0 otherwise

Table S2: **Description of the used datasets**

Sample	Platform	Accession number	Avg. read length	Number of bases
A. thaliana Col-0–Cvi-0	Illumina	SRX1865253	2 × 249 bp	8.1 Gb
A. thaliana Col-0–Cvi-0	PacBio	SRX1715706	5.6 kb	15.7 Gb
O. sativa Suijing18	Illumina	SRR5880534	2 × 150 bp	23.6 Gb
O. sativa Suijing18	PacBio	SRR5877285	8.5 kb	26.4 Gb
A. thaliana L*er*	Illumina	SRR3166543	2 × 100 bp	20.8 Gb
A. thaliana L*er*	PacBio	SRX533607	4.8 kb	36.1 Gb
Z. mays ssp. *mays* L. B73	Illumina	SRR2960981	2 × 250 bp	209.8 Gb
Z. mays ssp. *mays* L. B73	PacBio	SRX1472849	8.1 kb	279.3 Gb
S. lycopersicum PI158760	Illumina	ERR418074	2 × 100 bp	35.9 Gb
S. lycopersicum LA2706	Illumina	ERR418039	2 × 100 bp	31.4 Gb
S. lycopersicum TR00003	Illumina	ERR418051	2 × 100 bp	33.3 Gb
S. lycopersicum LA4451	Illumina	ERR418065	2 × 100 bp	29.1 Gb
S. arcanum LA2157	Illumina	ERR418092	2 × 100 bp	32.0 Gb
S. habrochaites LYC4	Illumina	ERR418105	2 × 100 bp	31.8 Gb
S. pennellii LA0716	Illumina	ERR418107	2 × 100 bp	24.9 Gb

Table S3: **Number of events called from B73 data that could not be validated by VaPoR**

Tool	Number of events	% of total
Delly	18074	44.41
GRIDSS	43006	48.45
LUMPY	7635	24.08
Manta	6957	23.74
MetaSV	35234	28.56
Survivor	2658	32.22
Parliament2	12869	34.12
Hecaton	54322	51.13

Table S4: **Number of events simulated per size interval**

Size interval	Number of events
100-200 bp	10
200-500 bp	10
500-1000 bp	10
1-2 kb	10
2-5 kb	10
5-10 kb	5
10-20 kb	5
20-50 kb	3
50-100 kb	3
